# Rise of oceanographic barriers in continuous populations of a cetacean: the genetic structure of harbour porpoises in Old World waters

**DOI:** 10.1186/1741-7007-5-30

**Published:** 2007-07-25

**Authors:** Michaël C Fontaine, Stuart JE Baird, Sylvain Piry, Nicolas Ray, Krystal A Tolley, Sarah Duke, Alexei Birkun, Marisa Ferreira, Thierry Jauniaux, Ángela Llavona, Bayram Öztürk, Ayaka A Öztürk, Vincent Ridoux, Emer Rogan, Marina Sequeira, Ursula Siebert, Gísli A Vikingsson, Jean-Marie Bouquegneau, Johan R Michaux

**Affiliations:** 1 MARE – Laboratory for Oceanology, University of Liège, Bat B6c, Liège (Sart Tilman) 4000, Belgium; 2 INRA, UMR CBGP (INRA/IRD/Cirad/Montpellier SupAgro), Campus international de Baillarguet, CS 30016, F-34988 Montferrier-sur-Lez cedex, France; 3 Computational and Molecular Population Genetics Laboratory, Zoological Institute, University of Bern, Switzerland; 4 Marine Mammal Division, Institute of Marine Research, Bergen, Norway; 5 Molecular Systematics Laboratory, South African National Biodiversity Institute, Private Bag X7, Claremont 7735, Cape Town, South Africa; 6 Department of Zoology, University College, Dublin, Ireland; 7 Laboratory of Biotechnological Research in Ecology, Medicine and Aquaculture (BREMA), Simferopol, Ukraine; 8 Portuguese Wildlife Society Estação de Campo de Quiaios. Apt 16 EC Quiaios. 3081-101 Figueira da Foz, Portugal; 9 Department of Pathology, Veterinary College, Sart Tilman B43, University of Liège, 4000 Liège, Belgium; 10 Coordinadora para o Estudio dos Mamiferos MAriños, CEMMA, Gondomar, Spain; 11 Faculty of Fisheries, Istanbul University, Ordu Cad. 200, Laleli-Istanbul, Turkey; 12 Centre de Recherche sur les Mammifères Marins, Institut de la Mer et du Littoral, Avenue du Lazaret, Port des Minimes, 17000 La Rochelle, France; 13 Department of Zoology, Ecology and Plant Science, University College, Cork, Ireland; 14 Instituto da Conservação da Natureza, Rua de Santa Marta, 55, 1150-999 Lisboa, Portugal; 15 Forschungs- und Technologie Zentrum, Westküste, Universität Kiel, Hafentörn 1, 25761 Büsum, Germany; 16 Marine Research Institute, Skúlagata 4, P.O. Box 1390, 121 Reykjavík, Iceland; 17 Génétique des Microorganismes, Département des Sciences de la Vie, Institut de Botanique B22, Université de Liège, 4000 Liège, Belgium

## Abstract

**Background:**

Understanding the role of seascape in shaping genetic and demographic population structure is highly challenging for marine pelagic species such as cetaceans for which there is generally little evidence of what could effectively restrict their dispersal. In the present work, we applied a combination of recent individual-based landscape genetic approaches to investigate the population genetic structure of a highly mobile extensive range cetacean, the harbour porpoise in the eastern North Atlantic, with regards to oceanographic characteristics that could constrain its dispersal.

**Results:**

Analyses of 10 microsatellite loci for 752 individuals revealed that most of the sampled range in the eastern North Atlantic behaves as a 'continuous' population that widely extends over thousands of kilometres with significant isolation by distance (IBD). However, strong barriers to gene flow were detected in the south-eastern part of the range. These barriers coincided with profound changes in environmental characteristics and isolated, on a relatively small scale, porpoises from Iberian waters and on a larger scale porpoises from the Black Sea.

**Conclusion:**

The presence of these barriers to gene flow that coincide with profound changes in oceanographic features, together with the spatial variation in IBD strength, provide for the first time strong evidence that physical processes have a major impact on the demographic and genetic structure of a cetacean. This genetic pattern further suggests habitat-related fragmentation of the porpoise range that is likely to intensify with predicted surface ocean warming.

## Background

In the marine realm, pelagic species that have large geographic range and high dispersal capabilities represent a serious challenge to the idea of allopatric divergence (i.e., a large continuous population broken up into smaller units by extrinsic barriers) and to speciation processes in a seemingly continuous environment [1]. The high mobility of these species and the dearth of barriers to gene flow in oceans might be expected to limit the division of species' ranges and, as a result, even distant regions might be connected genetically [1,2]. Although examples of genetic homogeneity over large distances are common in marine systems, there are also many examples of surprising population structure in marine species with high dispersal potential [1,3-7].

Cetaceans are good examples of this kind of species. Despite their broad range and their high dispersal capabilities, many cetaceans often show substantial genetic structure at regional or even fine scale, although the extent varies among species [8]. It is generally argued that these patterns, not always correlated with geographic features, are related to a combination of complex behaviours, such as philopatry, specialisations for local resources, or social organisation into kinship groups [8,9]. On the other hand, while the dispersal and segregation of populations of terrestrial mammals are frequently influenced by geographic features or climatic characteristics, few such obvious barriers are expected to restrict cetacean dispersal and gene flow in the world's oceans [10,11]. Variation in oceanographic properties of the water column, such as depth, temperature, currents and winds, are known as important factors in the life of these animals, most obviously in conditioning the availability of their food (for example, see [12]), but their effect on cetacean dispersal and on population structure remains enigmatic.

**Figure 1:**
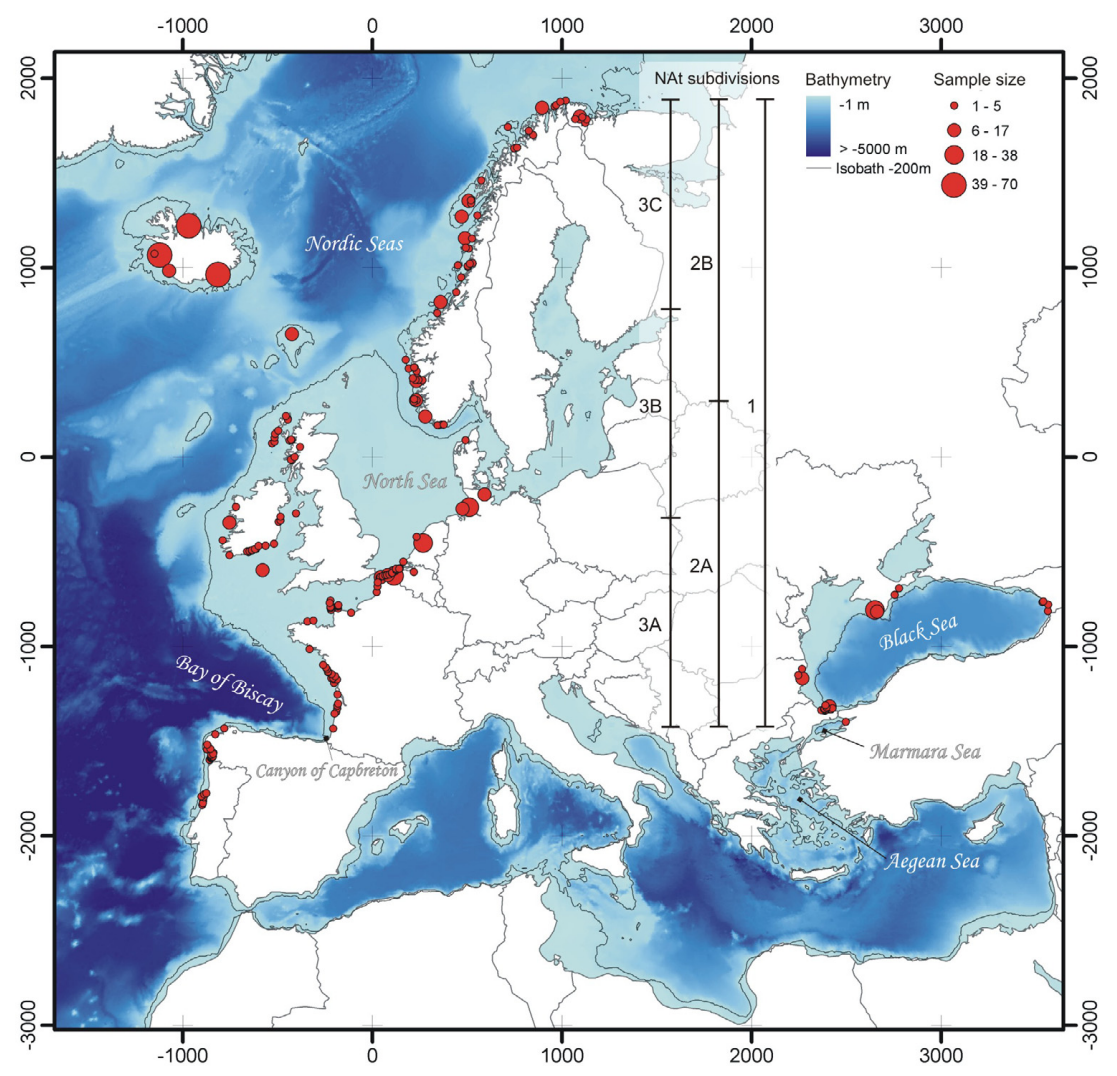
**Bathymetric map of the eastern North Atlantic showing the approximate geographic sampled locations and sample sizes per location**. Geographic locations are based on GPS coordinates or reported discovery location. The bar scales delimit the latitudinal range of the three spatial scales considered for the analyses of the North Atlantic (NAt) cluster: the global scale (1); the middle scale, south (2A), and north (2B) parts; and the small scale, the south (3A), middle (3B), and north (3C) parts. The map is projected using a gnomonic projection centred on the sampling centroid (scale units in km).

Small coastal cetaceans such as those of the porpoise family are a model of choice to investigate this issue because they have to face a suite of intrinsic problems not encountered by larger dolphins and whales. Their small size, their demanding reproductive schedule, and their limited ability to store energy force a strong dependency on their food [13,14]. Therefore, we expect that variation in oceanographic features that determine food availability and abundance (i.e., bathymetry, temperature and primary productivity) should markedly affect local density and dispersal of porpoises. If true, their population genetic structure should correlate, at least partly, with oceanographic characteristics. To test this hypothesis, we examined the genetic structure of one the most widely distributed porpoises, the harbour porpoise *Phocoena phocoena *(L. 1758), with regards to seascape characteristics. Harbour porpoises occur fairly continuously throughout cold coastal waters of the North Pacific and the North Atlantic, with a relict population in the Black Sea separated from the Atlantic range by the Mediterranean Sea where porpoises are nowadays absent [15-17]. We analysed genetic polymorphism at 10 microsatellite loci for an extensive sampling (n = 752) covering the main distribution of harbour porpoises in the central and eastern North Atlantic (Figure 1) using a combination of recent individual-based landscape genetic approaches [18-21].

Here, we provide strong and clear evidence that seascape imposes major constraints on the demographic and genetic structure of a cetacean, and thus on its dispersal. This finding is of general interest in the context of climate change and habitat fragmentation for marine species, as ecosystems in the eastern North Atlantic are shifting toward a warmer dynamic equilibrium with significant changes already detected in plankton and fish assemblages.

## Results

We applied two complementary Bayesian clustering algorithms, namely *Structure *v.2.1 [18,19] and *Geneland *v.1.0.7 [20], to infer population structure (i.e., a number of clusters, *K*) and to assign individuals (probabilistically) to populations (or clusters) based on individual multilocus genotypes and, for the second algorithm, also on individual spatial origins. Both of these approaches assume that populations are panmictic units with distinct allele frequencies. To test whether individual dispersal is restricted in space, we analysed the pattern of isolation by distance (IBD) using the individual-based approach developed by Rousset [21]. This involves regression of an index of genetic differentiation on marine geographic distance among pairs of individuals (see Methods). Finally, recent migration among populations (within the last few generations) was assessed using a Bayesian model implemented in *BayesAss *v.1.3 [22]. This algorithm requires few assumptions for assigning individual genotypes to population of origin and, in particular, relaxes the key assumption of Hardy-Weinberg (HW) equilibrium within populations.

### Clustering analyses

#### Structure analysis

**Figure 2:**
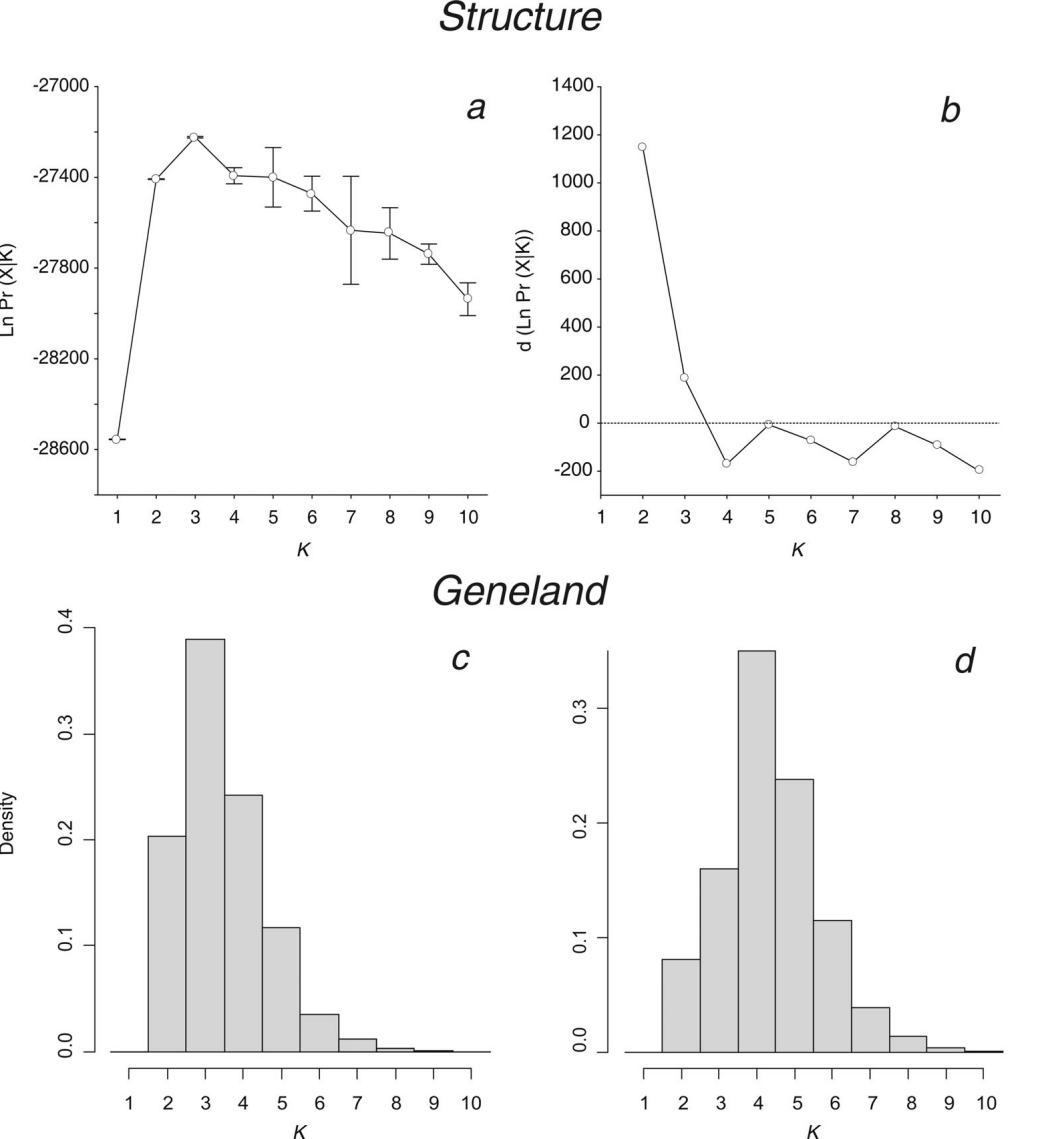
**Estimated number of populations from Structure (a and b) and Geneland (c and d) analyses**. *Structure *analyses: (a) mean (± SD) probabilities of the data [LnPr(X|*K*)] over 10 *Structure *replicated runs plotted as a function of the putative number of clusters (*K*). (b) Mean variations of probabilities of the data (Δ(LnPr(X|*K*)) between successive *K *considered in *Structure *analyses. For *K *clusters, this variation is calculated as Δ(LnPr(X|*K*))=LnPr(X|*K*)_*k*+1_-LnPr(X|*K*)_*k*_. *Geneland *analyses: posterior density distribution of the number of clusters estimated from *Geneland *analysis in 7 out of 10 replicates (c) and in the 3 remaining trials (d).

**Figure 3:**
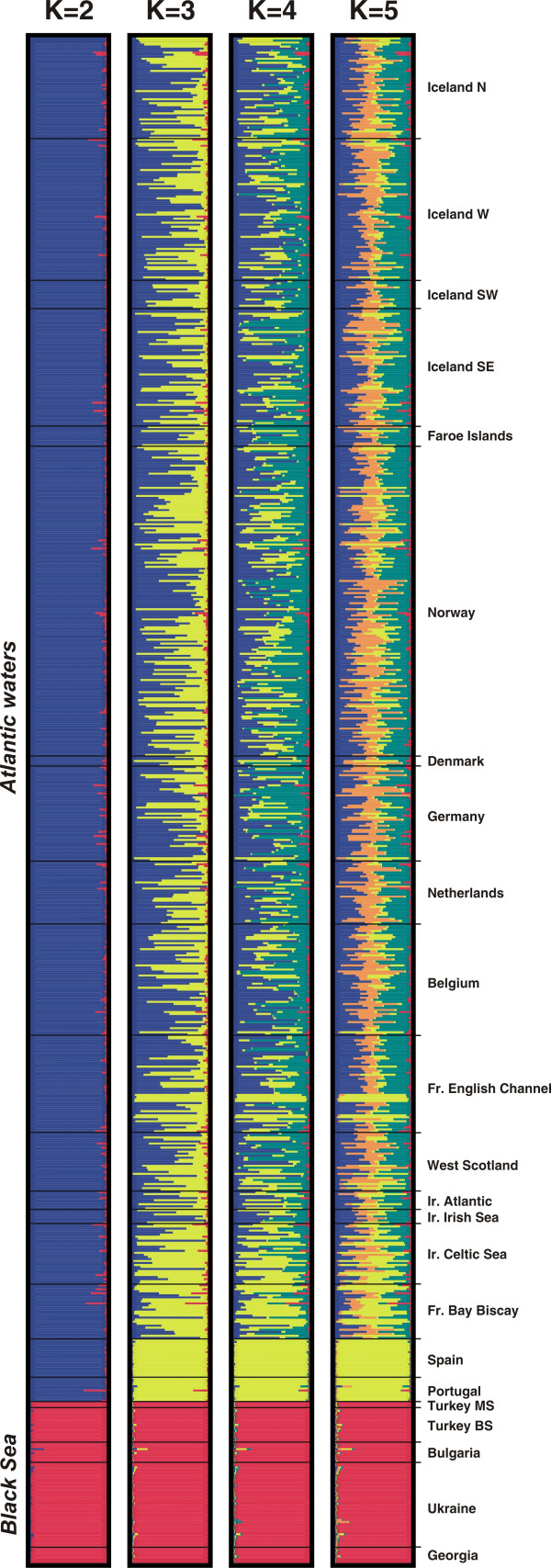
**Estimated population structure from Structure analyses for *K *= 2 to *K *= 5**. Each individual is represented by a thin horizontal line divided into *K *coloured segments that represent the individual's estimated membership fractions in *K *clusters. Black lines separate individuals from different geographic areas labelled on the right. Each plot, produced with *Distruct *[79], is based on the highest-probability run (of ten) at that value of *K*. Individuals are arranged based on their origins and sorted with increasing latitude.

*Structure *provided consistent results over 10 replicated runs tested for each *K *and over the different models tested (see Methods). Generally, in highly structured data sets, as *K *is increased the most divergent groups separate into distinct clusters first [18,23]. The probability of the data (LnPr(X|*K*)) greatly increased from *K *= 1 to *K *= 2, and then reached a maximum value at *K *= 3, after which the values decreased gradually (Figure 2a). The increase of likelihood (Δ(LnPr(X|*K*)); Figure 2b), i.e. the gain of explanatory power of the model when adding a new cluster to the analysis, is high at *K *changing from 1 to 2. At *K *= 2, the two clusters are anchored by the Black Sea (BS) and the North Atlantic porpoises (Figure 3). The addition of a third cluster (*K *= 3) further increases the probability of the data, the gain of power becoming null or negative for higher values of *K *(Figure 2b). At *K *= 3, the North Atlantic cluster splits in two distinct parts that persist and become more clearly distinct for higher values of *K *(Figure 3). The first is a genetically homogeneous cluster that encompasses porpoises from Spain and Portugal with high membership coefficients (Iberian cluster, IB). The second group is composed of the remaining individuals sampled further north (North Atlantic cluster, NAt). Most of these display membership coefficients that tend to distribute evenly across clusters others than the Black Sea and Iberian clusters as *K *is increased. The same pattern was observed whatever the model considered in the analysis. This pattern might result from (a) lack of sufficient signal in the data set to confidently assign these individuals, and/or (b) low underlying genetic structure of porpoises in that area, or (c) departure from the basic assumptions of the model. Instead of discrete genetic units at HW and linkage equilibrium, the population structure in northern waters might be much more continuous than discrete, with continuous gradations in allele frequency over the range (see below).

#### Geneland analysis

While *Structure *uses only the individual multilocus genotype data to infer the population structure, *Geneland *also exploits the spatial positions of the individual samples as a supplemental parameter in the analysis [20]. Interesting features of the *Geneland *model that further distinguish it from that of the *Structure *model are its ability (a) to deal with an unknown number of populations simultaneously with other parameters, (b) to locate population boundaries across space, and (c) to account for uncertainty in the positioning of sampled individuals (see Methods and [20] for further details). This last feature is particularly useful in the present context as the locations of sampled harbour porpoises, composed of by-caught and stranded animals, might be poorly representative of the normal range of individuals.

**Figure 4:**
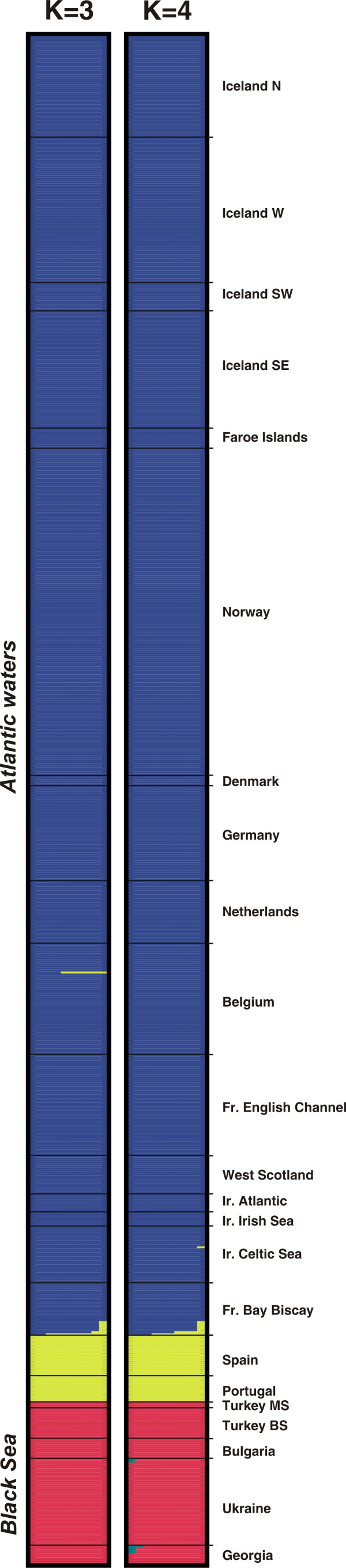
**Estimated population structure from Geneland analyses for the two modal solutions *K *= 3 and *K *= 4**. Each individual is represented by a thin horizontal line divided into *K *coloured segments that represent the individual's estimated membership fractions in *K *clusters. Black lines separate individuals from different geographic areas labelled on the right. Each plot, produced with *Distruct *[79], is based on the highest-probability run at that value of *K*. Individuals are arranged based on their origins and sorted with increasing latitude.

**Figure 5:**
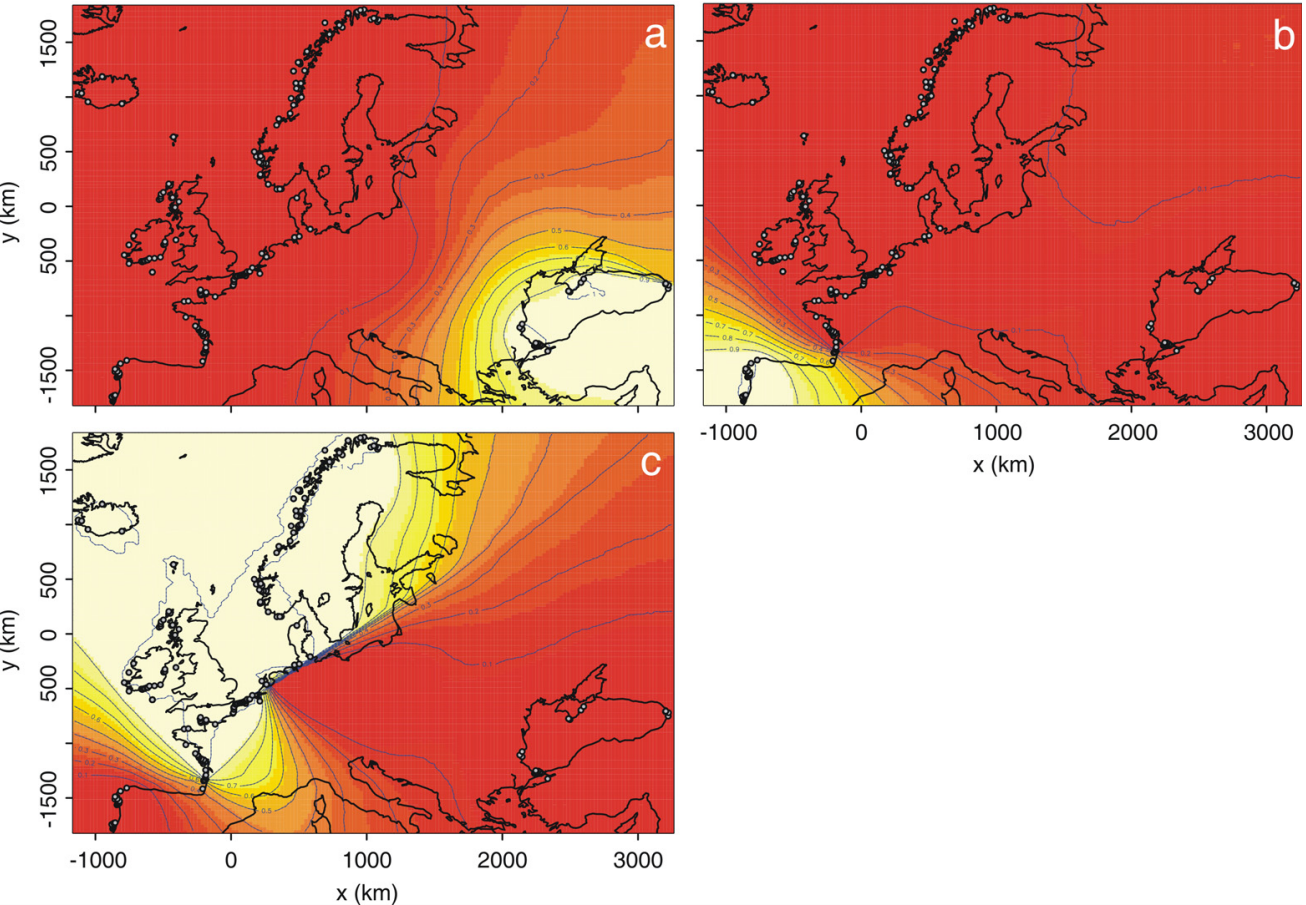
**Maps of Geneland individual assignments to clusters for *K *= 4 (scale units in km)**. The three plots represent the assignment of pixels to each cluster: (a) Black Sea cluster; (b) Iberian cluster; and (c) North Atlantic cluster. The assignments of pixels to the fourth cluster are not shown, as no individuals are assigned to it ("ghost cluster", see text for further details). The highest membership values are in light yellow and the level curves illustrate the spatial changes in assignment values. The plot is based on the highest-probability run at that value of *K*.

The *Geneland *model provided results consistent with those of the *Structure *one. Posterior distributions of the estimated number of populations (*K*) across 10 replicates displayed a clear mode at *K *= 3 in 7 out of the 10 replicates (Figure 2c) and at *K *= 4 in the remaining trials (Figure 2d). Similar to the *Structure *results, *Geneland *identified three spatially coherent clusters (Figures 4 and 5): the first gathers all porpoises from the Black Sea and Marmara Sea (the BS cluster) isolated from those in the Atlantic by the Mediterranean (Figure 5a); the second gathers the porpoises from the Iberian peninsula (the IB cluster) isolated from samples further north by a barrier to gene flow located in the southern Bay of Biscay (Figure 5b); and the third is unequivocally composed of the samples further north in the Atlantic (the NAt cluster), widely distributed from the French coast of the Bay of Biscay to the Arctic waters of Iceland and Norway (Figure 5c). This last result contrasts slightly with that of the *Structure *analysis (compare Figures 3 and 4). While the *Structure *model did not confidently assign these individuals, *Geneland *assigned almost all them to the NAt cluster with high membership coefficients that remain consistent even for higher values of *K *(Figure 4; *K *= 4). This suggests that taking into account the spatial context of individuals might improve the efficiency of the analysis. No individuals were assigned to the fourth cluster detected in 3 out of the 10 *Geneland *replicates (Figure 4, *K *= 4, green colour). This is not surprising as this cluster is centred on landmass (not shown). Such occurrences of "ghost" populations, with no individuals assigned, is reported by *Geneland*'s authors as a poorly understood problem [20]. It could be related to the process of tiling a heterogeneous sampling distribution, with "landmass" tiles being reported as a "ghost" population. As there are no individuals assigned to this cluster, as it only occurs in a minor proportion of the trials and as it does not affect biological interpretation in the present context, this "ghost" population can be ignored (as suggested by the *Geneland *authors [20]).

### Genetic diversity and differentiation among inferred populations

The three identified clusters differed greatly with respect to their genetic diversity assessed using heterozygosity and allelic richness, corrected for difference in sample size (Table 1). Harbour porpoises from Iberian waters and the Black Sea displayed comparable genetic diversity that was much lower than that observed in the NAt cluster. For example, the allelic richness over all loci was twice as low in the Black Sea and in Iberia as it was in the NAt cluster (Wilcoxon paired-sample test: BS-IB: p = 0.878; IB-NAt: p < 0.005; BS-NAt: p < 0.005).

**Table 1:** Genetic variation at the 10 microsatellite loci for populations inferred from the cluster analyses

	Black Sea				Iberia				North Atlantic			
Locus	n	A	*H*_*o*_/*H*_*e*_	*F*_IS_	n	A	*H*_*o*_/*H*_*e*_	*F*_IS_	n	A	*H*_*o*_/*H*_*e*_	*F*_IS_

415–416	77	2.7	0.39/0.44	0.115	29	2.0	0.24/0.22	-0.120	569	5.3	0.52/0.55	0.062***
EV94	78	3.3	0.47/0.48	0.009	29	5.0	0.65/0.66	0.001	576	6.5	0.76/0.79	0.035*
GATA053	78	1.4	0.01/0.01	-	31	2.0	0.42/0.34	-0.250	642	3.8	0.21/0.21	0.041**
GT011	78	2.7	0.45/0.41	-0.098	31	3.0	0.35/0.35	-0.019	642	9.5	0.82/0.82	-0.003
GT015	77	6.8	0.39/0.36	-0.075	29	16.0	0.90/0.91	0.092	553	18.9	0.87/0.94	0.076***
IgF-1	78	8.6	0.78/0.73	-0.072	31	4.9	0.26/0.29	0.119	642	11.5	0.83/0.87	0.043***
PPH104	77	7.1	0.67/0.65	-0.048	30	7.0	0.83/0.77	-0.085	638	12.5	0.86/0.88	0.031**
PPH110	77	5.4	0.48/0.50	0.046*	31	4.0	0.77/0.70	-0.111	641	9.1	0.77/0.82	0.068**
PPH130	78	5.9	0.60/0.65	0.077	30	7.9	0.60/0.66	0.087	642	12.7	0.80/0.89	0.106***
PPH137	78	6.6	0.73/0.66	-0.107	31	5.9	0.64/0.70	0.084	642	14.3	0.88/0.91	0.036**
Multilocus	78	5.1	0.50/0.49	-0.020	30	5.8	0.57/0.56	-0.016	619	10.3	0.73/0.77	0.050***

n: Sample size; A: allelic richness (estimated for a sample size of 29 individuals); *H*_*o*_: observed heterozygosity; *H*_*e*_: gene diversity; *F*_IS _values calculated after Weir and Cockerham [24]. Asterisks refer to the significance level of the tests for heterozygosity deficiency (*, p < 0.05; **, p < 0.01; ***, p < 0.001).

**Figure 6:**
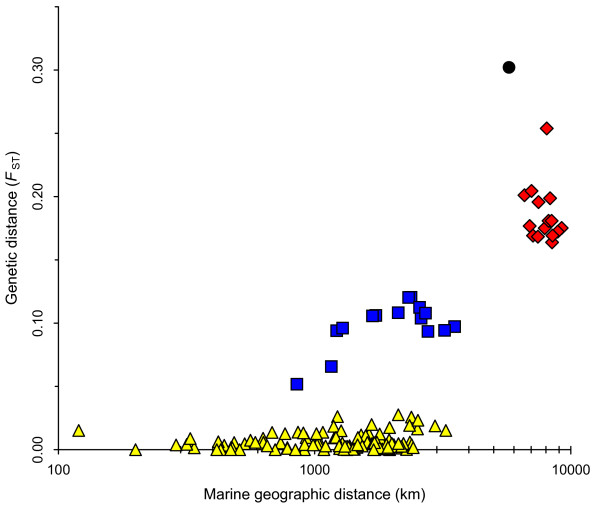
**Genetic and geographic distance for pairs of sampled geographic areas**. Yellow triangles indicate comparison between pairs of sampled localities within the same cluster; blue squares indicate pairs with one sampled locality in the NAt cluster and the IB cluster; red diamonds indicate pairs with one sampled locality in the NAt cluster and the BS cluster; and black circle indicate the comparison between the IB and the BS cluster.

The amount of genetic differentiation among clusters, estimated using *F*_ST _[24], illustrated the high divergence of Black Sea harbour porpoises from those in the North Atlantic (*F*_ST_: BS-IB = 0.314, 95% Confidence Interval (CI): 0.240–0.381; BS-NAt = 0.147, 95% CI: 0.116–0.179). The *F*_ST _values between Iberian porpoises and those sampled further north in the Atlantic were lower, but remained substantial (*F*_ST_: IB-NAt = 0.090, 95% CI: 0.054–0.131). In contrast, *F*_ST _values between parts of the NAt cluster (Figure 1 3A–C: ) were much lower (*F*_ST _≤ 0.001; see Additional file 1). Figure 6 provides a global view of the system. It shows that for pairs of sampled localities from different clusters, genetic differentiation is much larger than that between intracluster pairs that have the same geographic distance. In other words, genetic differentiation between clusters is not only induced by geographic distance between them but also by barriers to gene flow.

Tests of departure from HW equilibrium (Table 1) show no significant deviation for porpoises from Iberia and the Black Sea, but a significant deficiency in heterozygosity at 9 of the 10 loci analysed in the NAt cluster. This slight heterozygote deficiency recorded at almost all loci in porpoises of northern Atlantic waters and the failure of the *Structure *model to assign these individuals in comparison to the *Geneland *model suggest that a subtle spatial structure (i.e., Wahlund effect) with a continuous gradation in allele frequencies across regions and/or isolation by distance could occur [23,25].

### Isolation by distance analyses

When IBD occurs in 'continuous' populations distributed in a two-dimensional habitat, genetic differentiation among individuals is expected to increase linearly with the logarithm of geographic distance [21,26]. This linear relationship was demonstrated to hold best at local geographical scale because heterogeneity of demographic parameters (i.e., dispersal and/or density) and the effect of mutation rate are reduced and hence their confounding influence on genetic differentiation is also reduced [27,28]. However, the scale of population ranges in the marine realm is often unknown and can be quite large (of the order of hundreds or thousands of kilometres squared), especially for cetacean species [29]. As we cannot know the appropriate scale *a priori *for the NAt cluster, we conducted the IBD analyses considering the range at three different spatial scales (Figure 1 and Table 2). We first analysed IBD in the global range of the NAt cluster that latitudinally extends over 3237 km from the French coast of the Bay of Biscay northwards to the arctic waters of Norway and Iceland (global scale). Then, we subdivided the global range into two parts of equal latitudinal range (Medium scale: NAt-2A and B), then into three parts (Small scale: NAt-3A-C) and repeated the analysis on each part.

**Table 2:** Isolation by distance within the North Atlantic cluster (NAt). Three different scales defined from the latitudinal subdivision of the global range in two (Medium scale) and in three (Small scale) parts were analysed. See Figure 1 for the delimitations of the NAt subdivisions.

**Scale**	**n**	**Mean (max) marine distance (km)**	**Slope ± SE**	**Intercept ± SE**	**p-Value**	**4π*Dσ ***^2 ^(1/slope)
Global:	654	1523.8 (4393.2)	0.0037 ± 0.0015	0.0011 ± 0.0148	0.002	270.3
Medium:						
NAt-2A	289	824.4 (2639.9)	0.0080 ± 0.0025	-0.0566 ± 0.0293	0.001	125.0
NAt-2B	365	1171.1 (2901.1)	0.0028 ± 0.0018	0.0153 ± 0.0154	0.024	357.1
Small:						
NAt-3A	210	616.8 (1614.0)	0.0100 ± 0.0031	-0.0867 ± 0.0347	< 0.001	100.0
NAt-3B	141	713.3 (2029.1)	0.0025 ± 0.0025	0.0244 ± 0.0292	0.204	400.0
NAt-3C	303	1100.0 (2901.1)	0.0030 ± 0.0020	0.0167 ± 0.0174	0.043	333.3

We found a significant positive relationship between the index of genetic differentiation (*a*_*r*_) and the marine geographic distance among porpoises in the NAt cluster at all scales considered (Table 2) except one: the region NAt-3B. This latter corresponds to the area where the sample size is the lowest (n = 141), where the sampling is the most spatially heterogeneous (Figure 1), and also where the marine distances among porpoises are the shortest (Table 2). Therefore, the absence of significant evidence in this region likely results from the low power of the analysis to detect IBD (see, for example, [30]).

Rousset [21,26] demonstrated that the regression slope is proportional to 1/4π*Dσ *^2^, where *D *is the effective density of individuals and σ^2 ^the second moment of axial dispersal distance, best described as the mean squared parent-offspring axial dispersal distance. σ^2 ^can be understood as a measure of the speed at which two gene lineages issuing from an ancestor move away from each other, as it is the rate at which the mean squared axial distance between these two lineage increases per time unit [30]. The comparison among subset areas at the medium and at small scale showed significant north-south variation in the parameters of the regression for the 10 microsatellite loci (Table 2). The slope (or 1/4π*Dσ *^2^) in the south part of the NAt cluster was significantly higher than that in northern parts at medium scale (Wilcoxon paired-sample test, 2A-2B: p = 0.037) and at small scale (Wilcoxon paired-sample test, 3A-3B: p = 0.005; 3A-3C: p = 0.046; 3B-3C: p = 0.399), suggesting that either density (*D*), dispersal (σ^2^), or both are reduced in the south part compared to the north.

### Recent migration rates among populations

Recent migration rates (i.e., within the last few generations) were estimated between porpoises from the Black Sea, Iberia and the southern part of the NAt cluster (NAt-3A) adjacent to the detected barrier to gene flow (Table 3) using the *BayesAss *v.1.3 algorithm [22]. When simulating the effect of having no information in the data from which to estimate migration rates, we obtained a 95% CI of 0.675–0.992 for the proportions of individuals derived from the source populations each generation (or non-migrant rates) and a CI of 0.001–0.261 for migration rates. Confidence intervals recovered from the data set were considerably smaller than those obtained from the null hypothesis (Table 3), suggesting that the data set contained an appreciable amount of information to support the results.

**Table 3:** Mean ± SD (95% CI) posterior distributions for migration rates among harbour porpoise populations. Values along the diagonal (bold) are the proportion of individuals derived from the source population (or non-migrant) each generation.

	Migration rate from		
	
To	Black Sea	Iberia	NAt-3A
Black Sea	**0.996 ± 0.004**	0.002 ± 0.003	0.002 ± 0.003
(n = 78)	**(0.984–1)**	(0–0.009)	(0–0.010)
Iberia	0.010 ± 0.011	**0.978 ± 0.017**	0.011 ± 0.011
(n = 31)	(0–0.041)	**(0.935–1)**	(0–0.042)
NAt-3A	0.003 ± 0.003	0.031 ± 0.012	**0.965 ± 0.013**
(n = 303)	(0–0.012)	(0.008–0.057)	**(0.938–0.988)**

Virtually all porpoises from the Black Sea were identified as non-migrant (Table 3). Although this result is not surprising, as the Black Sea population is now geographically isolated from the Atlantic populations by the Mediterranean Sea, this result can be useful as reference to assess the status of the Iberian population. Almost all porpoises from Iberian waters were also identified as non-migrant (98% of the individuals and the 95% CI upper limit including 1), while the NAt cluster showed a slightly lower non-migrant proportion (96%; Table 3). The migration rates between Iberia and the NAt cluster were low (m ≤ 0.03) with the lower 95% CI bounds not different from 0, except in one case: the migration rate from Iberia to the NAt cluster appeared slightly higher than the reverse, but the large overlap of 95% CIs did not allow us to conclude there was asymmetry in migration rates.

## Discussion

The individual-based approaches we used here revealed that most of the harbour porpoise range in the central and eastern North Atlantic behaves as a 'continuous' population that widely extends over thousands of kilometres from the French coasts of the Bay of Biscay northwards to the arctic waters of Norway and Iceland, with significant isolation by distance. This striking result is concordant with the low but sometimes significant level of genetic differentiation previously reported at microsatellite loci between arbitrarily defined groups in the North Sea and adjacent waters [31,32]. However, strong barriers to gene flow in the south-eastern North Atlantic range isolate, on a relatively small scale, porpoises from Iberian waters and on a larger scale porpoises from the Black Sea.

The total isolation of harbour porpoises from the Black Sea has long been suggested on the basis of the lack of field observation of porpoises in the Mediterranean Sea [17], of private mtDNA alleles reported in that population [33], and of morphological differences [34]. Our results lend further support to this hypothesis. The pronounced genetic footprint of this isolation left at nuclear and mtDNA loci suggest this is an ancient isolation that might date back to the last Ice Age ([35] and Fontaine, unpublished results). The genetic differentiation detected at microsatellite loci between the Iberian porpoises and those further north was not apparent at the mtDNA control region previously analysed [35]. The lack of mitochondrial lineage sorting and of private microsatellite alleles suggests that the differentiation we observed with microsatellite analyses is caused by a more recent isolation process than that of the Black Sea.

**Figure 7:**
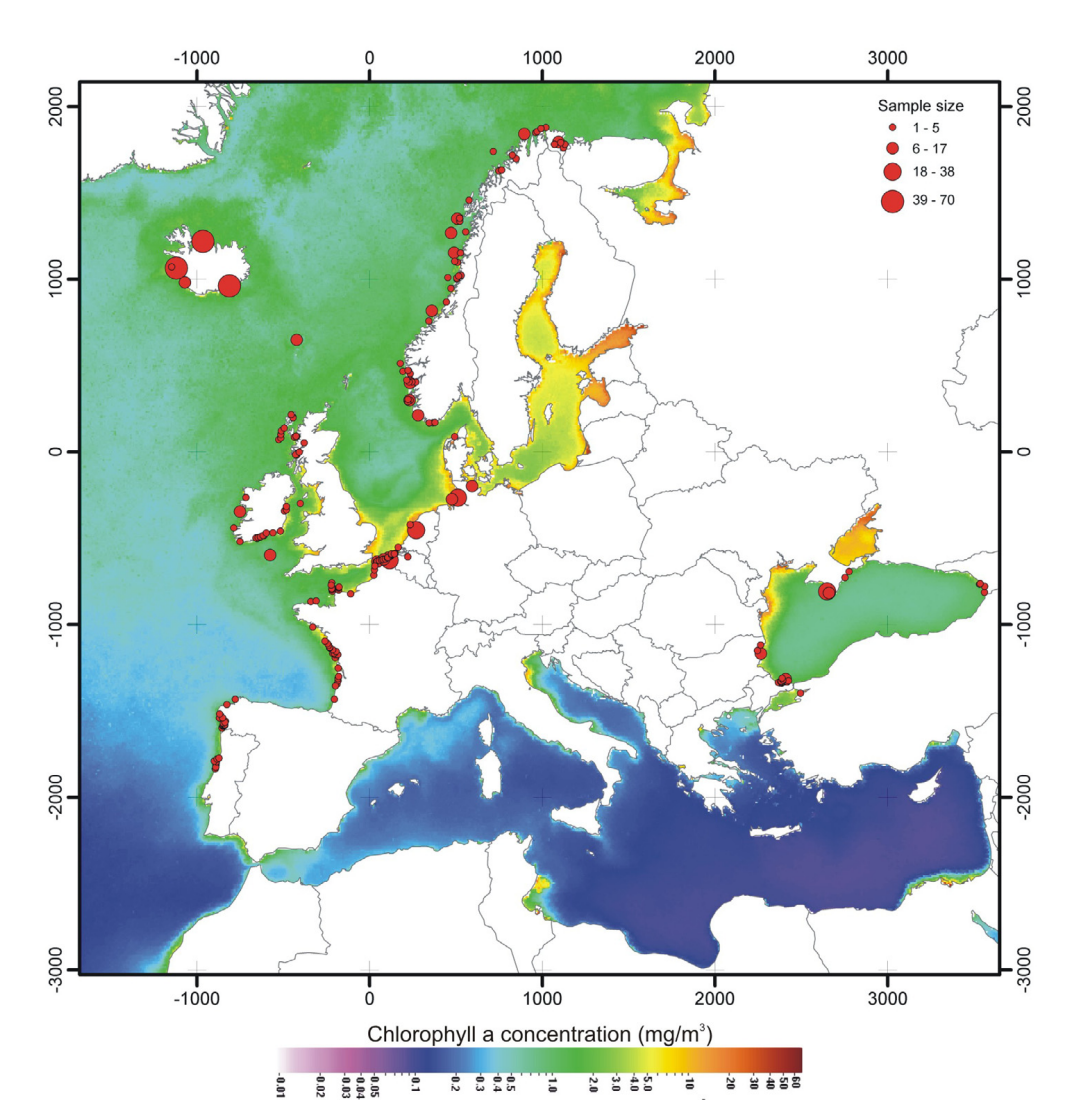
**Climatological (1997–2006) annual sea surface chlorophyll concentrations**. Data obtained with Sea-viewing Wide Field-of-view Sensor (SeaWIFS, modified from [80]).

The corollary of these results is the inference of strong barriers to gene flow in the southern Bay of Biscay and in the Mediterranean Sea that isolate almost completely the Iberian and Black Sea populations. These barriers coincide with strong oceanographic changes of similar nature (compare Figure 5 with Figures 1 and 7). To take them in turn, the conditions in the southern Bay of Biscay differ sharply from those at its margins [36,37]. The continental shelf, widely extended in the northern part, narrows considerably to the south and is cleaved asunder by the Cap Breton canyon, which drops to the abyssal plain in the south-east, only 10 km from the shore. Warm and oligotrophic surface water spreads from the Cap Breton canyon to cover half of the southern Bay in summer [36,37]. In contrast, off the Iberian Atlantic coast upwelling becomes evident from late spring to early autumn [38], bringing to the surface cold nutrient-enriched waters that support a rich food-web [39]. On the north side of the barrier, shallow, cold, and nutrient rich waters prevail most of the year from the French waters of the Bay of Biscay northward to the northern North Sea. From a biogeographical point of view, the southern Bay of Biscay is not only a barrier for porpoises but it is also a transition zone between the boreal and subtropical provinces, with many species reaching their southern or northern limit of distribution in that area [40].

Still further north, depth increases towards Nordic Seas (Figure 1), but waters remain cold and highly productive [41]. However, the bathymetric change does not seem to restrict gene flow in Nordic Seas, consistent with sightings of some porpoises reported far offshore in deep water [42]. While this suggest there are few, if any, potential barriers to dispersal of porpoises from the northern Bay of Biscay up to Arctic waters, the heterozygosity deficit related to the detected IBD shows nevertheless that porpoises do not mate randomly over that extended area and that gene flow is spatially restricted. We observed a north-south variation in the IBD pattern with higher IBD slope at the southern end of this range compared to northern parts (Table 2). One could argue that this north-south variation in IBD pattern might reflect drift disequilibrium [43] in northern areas associated with the postglacial porpoise recolonisation of Nordic waters in contrast to the southern habitats, which likely remained more stable in time. However, simulation-based sensitivity analysis of current *Dσ *^2 ^estimation to demographic instability in time and space conducted by Leblois et al [44] showed that spatial expansion with constant density does not significantly affect present-time *Dσ *^2 ^estimation, especially when the spatial expansion occurred 20 or more generations ago, as it is the case for postglacial recolonisation. Consequently, the higher IBD slope detected in the southern area (NAt-3A) compared to that in waters further north (NAt-3B and NAt-3C) most likely represents a lower current-time *Dσ *^2^. Although we cannot exclude variation in σ^2^, a lower porpoise density in southern waters is supported by field estimates based on aerial and ship surveys conducted in the North Sea and adjacent waters [45,46]. These variations in density (and maybe in dispersal patterns) likely reflect variation in habitat. The southern part of the 'continuous' population (i.e., the northern part of the Bay of Biscay, the English Channel and the southernmost part of the North Sea) borders the barrier to gene flow detected in the southern Bay of Biscay and should thus display sub-optimal conditions for porpoises while the middle (i.e., the central and northern North Sea) and northern areas (i.e., the Nordic Seas) would be more optimal for a cold water species such as the harbour porpoise.

The Mediterranean Sea displays similar characteristics to those encountered in the southern Bay of Biscay but at a larger scale. The Mediterranean is composed mostly of deep basins and narrow continental shelves with warm oligotrophic surface waters prevailing most of the year [47]. These characteristics are likely quite unfavourable for cold water species and might explain why the harbour porpoise is absent from this area. The oceanographic conditions in the Black Sea are, by contrast, more suitable for harbour porpoises with low salinity, colder and more nutrient rich surface waters than in the Mediterranean Sea [48]. There are however reports of porpoise strandings in the northern Aegean Sea [17]. This can be understood with regard to oceanographic features in that area. The subdivision of the Aegean into two basins has long been recognised. The northern basin is under the influence of cold, low salinity waters that pour out of the Black Sea. This water is entrained into a cyclonic circulation affecting the northern and western parts of the Aegean, causing an ecological isolation of the northern basin from the southern basin [49]. In the southern basin the continental shelf is very limited and the waters become quickly characteristic of Mediterranean waters [50], unfavourable for harbour porpoises.

To summarise, surface water temperature and primary production seem to be the factors that best characterise the nature of barriers to gene flow encountered across the harbour porpoise range, their population structure, and their geographic distribution. It is worth noting however that in oceanography, these two parameters are often linked [51]. Indeed, the sea surface temperature acts as a useful proxy for other physical processes, such as vertical stratification and nutrient contents, regulating the size structure, taxonomic composition, and abundance of the phytoplankton community, and thus the food availability for top predators [52,53]. These results reinforce previous ecological studies on harbour porpoises that reported significant relationships between abundances and movements with sea surface temperature and food availability [54,55]. Although bathymetry can be important in harbour porpoise ecology [56,57], we showed that this factor alone seems not to restrict gene flow in northern waters of the sampling range.

While the proximal causes of porpoise dependence on these habitat characteristics are beyond the scope of this paper, the ultimate underlying mechanism is likely related to the high energetic constraints this small cetacean has to face in order to survive. As one of the smallest endothermic marine predators, and furthermore with limited energy storage capacity, it is currently assumed that harbour porpoises must feed frequently without prolonged periods of fasting [16,58]. Their distribution, their movements, and in sum their overall biology should therefore be closely related to those of their prey and thus to nutrient rich waters.

## Conclusion

In the marine realm, community structure is shaped heavily by physical processes (see, for example, [47,59]). In this study we provide for the first time strong evidence that physical processes determining food availability have major impacts on the demographic and genetic structure of a cetacean. The small body size of harbour porpoises undoubtedly has profound consequences at all levels of their biology and makes this species particularly sensitive to habitat variation. We can however reasonably expect that this will be also applicable to other cetaceans of similar body size, habitat and thermoregulation constraints. However, these constraints could be reduced for larger cetaceans, leading to more complex patterns of population structure not necessarily correlated to seascape features (see, for example, [11]). Ecosystems in the eastern North Atlantic are shifting toward a warmer dynamic equilibrium with significant changes detected in plankton and fish assemblages [51,60-62], but the consequences for marine mammals remain to date unclear [63]. Although further analyses would be require to address the demographic trends of these populations, the genetic pattern highlighted here (i.e., the ancient isolation of harbour porpoises in the Black Sea), the more recent isolation of those in Iberian waters, and the higher IBD in the southern end of the northern Atlantic continuum, suggests that habitat-related fragmentation of harbour porpoise range is under way and that it is likely to continue with the predicted changes in climate.

## Methods

### Sample collection

Tissue samples were taken from by-caught and stranded harbour porpoises. A total of 752 animals distributed along the eastern North Atlantic range of the harbour porpoise and in the Black Sea were analysed (Figure 1). Out of these, 515 samples were analysed in this study and 237 samples from Iceland and Ireland were analysed by Duke [64].

Most of the individuals were geo-referenced using GPS coordinates recorded at the time or deduced from the reported location where the animal was found. These coordinates are naturally rough approximations to the normal locations of animals, especially for stranded animals, but this error can be considered negligible at the scale of the study range. This source of error can also be taken into account in some of the spatial analyses (see below).

### DNA extraction and microsatellite analysis

Total genomic DNA was extracted from tissues using the DNeasy™ Tissue Kit (Qiagen) following the manufacturer's recommendations. Samples were genotyped at 10 microsatellite loci using the multiplex sets defined in [65]. Polymerase chain reaction conditions were as reported in [65]. Amplified DNA was analysed for length variations on an automated 96 capillary MegaBace-1000 DNA Analyser (Amersham Biosciences) using Genetic Profiler v.1.5 (Amersham Biosciences).

### Habitat characteristics

Data on habitat characteristics across the study range with respect to salinity and sea surface temperature were taken from the National Oceanographic Data Centre (NODC) [66]. Bathymetric data were extracted from the ETOPO2 dataset available on the US National Geophysical Data Centre (NGDC) [67] and the data on chlorophyll concentration were taken from the NASA Sea-viewing Wide Field-of-view Sensor database (SeaWIFS) [68].

### Clustering analyses

We applied two Bayesian model-based clustering algorithms to infer population structure and to assign individuals (probabilistically) to clusters without *a priori *knowledge of population units and limits.

### Structure procedure

The first approach, implemented in *Structure *v.2.1, uses individual multilocus genotype data to cluster individuals into *K *groups while minimising Hardy-Weinberg disequilibrium and gametic phase disequilibrium between loci within groups [18,19]. The estimation procedure consists of running trial values of the number of populations *K *and then comparing the estimated log probability of data under each *K*, Ln [Pr(X|*K*)]. We conducted a series of independent runs with different proposals for *K*, testing all values from 1 to 10. Each runs used 10^6 ^iterations after a burn-in of length 4 × 10^4^, testing different models: (a) with or without admixture, and (b) correlated or uncorrelated allele frequencies. To check for convergence of the Markov chain Monte Carlo (MCMC), we performed 10 replicates for each value of *K *and then checked the consistency of results. The estimated number of clusters (*K*) was taken to be the value of *K *with the highest Pr(X|*K*) [18].

### Geneland procedure

The second algorithm, implemented in *Geneland *v.1.0.7, differs from that of Pritchard et al [18] mainly by taking into account explicitly the spatial dependence of individuals expected for species whose range is much larger than the average intergeneration movement of individuals. This model aims at inferring and locating genetic discontinuities between populations in space from individual geo-referenced multilocus genotypes, while taking into account uncertainty in the location of sampled individuals [20,69]. All the parameters (including *K*) are co-estimated simultaneously by the MCMC algorithm. However, for technical reasons discussed in [20], it is better to proceed in two steps: a first run to infer *K*, and a second run with *K *fixed at the modal value to estimate the other parameters (mainly the assignment of individuals to the inferred populations). The first step was replicated 10 times to check for convergence, allowing *K *to vary from 1 to 10 clusters and using the following run parameters: 10^6 ^MCMC iterations, maximum rate of Poisson process fixed at 700, maximum number of nuclei in the Poisson-Voronoi tessellation fixed to 500, and an uncertainty associated with the spatial coordinates of 50 km. We used the Dirichlet model of allelic frequencies as it has been demonstrated to perform better than the alternative model [20]. We inferred the number of clusters (*K*) from the modal value of *K *for these 10 runs, and then ran the MCMC again 100 times with *K *fixed for this value, 5 × 10^5 ^MCMC iterations, and the other parameters unchanged. We calculated the mean logarithm of posterior probability of the data (PPD) for each of the 100 runs and selected the 10 with the highest PPD. These 10 runs were then post-processed (with a burn-in of 5 × 10^4 ^iterations) in order to obtain posterior probabilities of population membership for each individual and each pixel of the spatial domain (174 pixels along the X axis and 143 along the Y axis corresponding to a pixel size of 25 km side). We finally checked visually for the consistency of results across these 10 runs.

### Descriptive statistics among clusters

The allelic richness, corrected for difference in sample size, the observed (*H*_*o*_) and expected (*H*_*e*_) heterozygosity (or genetic diversity), and *F*_IS _values were calculated within each cluster using *Fstat *v.2.9.3 [70]. To test whether genetic diversity was significantly different between clusters, we applied a Wilcoxon paired-sample test [71] on the 10 single locus values of the statistics of interest.

Level of genetic differentiation at microsatellite loci among clusters was estimated as *F*_ST _after Weir and Cockerham [24] using *Fstat *v.2.9.3 [70]. The 95% confidence interval was calculated using 15000 bootstrap resamplings [70]. We conducted exact tests to assess deviations from Hardy-Weinberg equilibrium and test for population differentiation using *Genepop *v.3.4 [72].

### Isolation by distance analysis

In continuous populations, an isolation by distance pattern occurs when genetic differentiation among individuals increases with their geographic distance [73]. Here we consider the statistic *a*_*r*_, a multilocus estimator of an *F*_ST_/(1-*F*_ST_) analogue between pairs of individuals [21]. When a continuous population is represented by a two dimensional lattice (i.e., fixed individual positions and no spatial density heterogeneity), *a*_*r *_is approximately linearly related to the logarithm of the geographic distance between individuals (*r*), *a*_*r *_≈ (Ln(r)/4π*Dσ *^2^) + C, where *D *is the effective density of individuals, σ^2 ^is the second moment of the dispersal distance distribution, and C is the value of the linear approximation at *r *= 1 length unit. Values of *a*_*r *_were regressed against the log of the marine geographical distance (see below) between paired individuals, as described in Rousset et al [21]. Significance of the regression slope was tested by 10^5 ^random permutations of individual locations (similar to a Mantel test) using the computer program *SPAGeDi *v.1.2 [74]. Assuming low mutation rate, the inverse of the regression slope provides an estimate of the product 4π*Dσ *^2 ^[21,26]. To test whether the regression slopes significantly differed between the different parts of a same scale, we used a Wilcoxon paired-sample test [71] applied on the 10 single locus values of the regression slope.

In the marine realm, the Euclidean distance between individuals might not be representative of the effective geographic distance separating them. Therefore, we computed an effective marine geographic distance between individuals using the least-cost path (LCP) algorithm implemented in the *Pathmatrix *extension [75] of the geographical information system software *ArcView *v.3.X (ESRI, Redlands, CA, USA). This algorithm computes a deterministic LCP between a source point and a target point by using a friction (or resistance) layer. The friction layer is a raster map where each cell (landscape unit) expresses the relative difficulty (or cost) of moving through that cell. A LCP minimises the sum of costs of all cells along the path (for detailed description and discussion of the algorithm, see [76]). In the present study, a uniform cost was attributed to all sea cells, while land cells harboured an "infinite" cost. This allowed us to compute effective distances avoiding landmasses. The sea/land map was obtained by rasterising (at 2 km resolution) a polygon version [77] of the GSHHS shoreline dataset v.1.3 [78]. The computations were performed using a gnomonic projection around the centroid of the sampled localities, which minimises the map deformation in planar distances induced by the curvature of the earth (Baird personal communication). Finally, the length of pairwise LCP (in meters) was introduced as the geographic distance matrix separating pairs of individuals in the regression analyses described above.

### Migration rates among clusters

Evidence of recent migration events across clusters was assessed using the Bayesian multilocus genotyping procedure implemented with MCMC methods in *BayesAss *v.1.3 [22]. This approach does not require populations to be in either migration-drift or Hardy-Weinberg equilibrium. To examine the strength of the information in the porpoise microsatellite data set, 95% confidence intervals were determined for migration rates and compared to a scenario where all proposed changes throughout the Markov chain are accepted (thereby simulating the situation where any information that could exist in the data is insufficient to affect the posterior distribution of migration rates, as suggested by the authors). The MCMC was run for a total of 3 × 10^6 ^iterations, with the first 10^6 ^discarded as a burn-in to allow the chain to reach stationarity. Samples were collected every 2000 iterations to infer posterior probability distributions of parameters of interest.

## Authors' contributions

MCF conceived and designed the experiments with help from SJEB. MCF performed the laboratory experiments except the analysis of samples from Iceland and Ireland analysed by SD. MCF analysed the data and interpreted the results with help from SJEB, JRM, SP, and NR. NR conceived the algorithm to calculate the marine geographic distance used in the Isolation by distance analysis. SP provided cluster computation assistance for the data analyses. JMB and JRM provided logistical support for this study. KAT, AJB, MF, TJ, ÁL, BÖ, AAÖ, VR, ER, MS, US, GAV provided the biological materials for the study. MCF wrote the manuscript with help from SJEB. All authors read and approved the final manuscript.

## Supplementary Material

Additional file 1 **Levels of genetic differentiation at microsatellite loci estimated as *F*_ST _among the populations inferred from the cluster analyses**. The North Atlantic cluster (NAt) was subdivided latitudinally in three parts (see Figure 1). The *F*_ST _values [95% CI] are below the diagonal and the significance level of the exact tests for population differentiation [72] are above. Click here for file

## References

[B1] Palumbi SR (1994). Genetic divergence, reproductive isolation and marine speciation. Annu Rev Ecol Syst.

[B2] Thorrold SR (2006). Ocean ecology: don't fence me in. Curr Biol.

[B3] Barber PH, Palumbi SR, Erdman MV, Moosa MK (2000). A marine Wallace's line?. Nature.

[B4] Taylor MS, Hellberg ME (2003). Genetic evidence for local retention of pelagic larvae in Caribbean reef fish. Science.

[B5] Bekkevold D, Andre C, Dahlgren TG, Clausen LA, Torstensen E, Mosegaard H, Carvalho GR, Christensen TB, Norlinder E, Ruzzante DE (2005). Environmental correlates of population differentiation in Atlantic herring. Evolution.

[B6] Jorgensen HBH, Hansen MM, Bekkevold D, Ruzzante DE, Loeschcke V (2005). Marine landscapes and population genetic structure of herring (*Clupea harengus *L.) in the Baltic Sea. Mol Ecol.

[B7] Kenchington EL, Patwary MU, Zouros E, Bird CJ (2006). Genetic differentiation in relation to marine landscape in a broadcast-spawning bivalve mollusc (*Placopecten magellanicus*). Mol Ecol.

[B8] Hoelzel AR, Goldsworthy SD, Fleischer RC, Hoelzel AR (2002). Population genetics. Marine Mammal Biology: An Evolutionary Approach.

[B9] Hoelzel AR (1998). Genetic structure of cetacean populations in sympatry, parapatry, and mixed assemblages: implication for conservation policy. J Hered.

[B10] Palsbøll PJ, Clapham PJ, Mattila DK, Larsen F, Sears R, Siegismund HR, Sigurùnsson J, Vasquez O, Arctander P (1995). Distribution of mtDNA haplotypes in North Atlantic humpback whales: the influence of behaviour on population structure. Mar Ecol Prog Ser.

[B11] Natoli A, Birkun A, Aguilar A, Lopez A, Hoelzel AR (2005). Habitat structure and dispersal of male and female bottlenose dolphins (*Tursiops truncatus*). Proc R Soc B.

[B12] Berta A, Sumich JL (1999). Marine Mammals: Evolutionary Biology.

[B13] Read AJ, Perrin WF, Würsig B, Thewissen JGM (2002). Porpoises, overview. Encyclopedia of Marine Mammals.

[B14] Koopman HN, Pabst DA, McLellan WA, Dillaman RM, Read AJ (2002). Changes in blubber distribution and morphology associated with starvation in harbour porpoise (*Phocoena phocoena*): evidence for regional variation in blubber structure and function. Physiol Biochem Zool.

[B15] Gaskin DE (1984). The harbour porpoise *Phocoena phocoena *(L.): regional populations, status, and information on direct and indirect catches. Rep Int Whal Commn.

[B16] Read AJ, Ridgway SH, Harrison R (1999). Harbour porpoise (*Phocoena phocoena*). Handbook of Marine Mammals.

[B17] Frantzis A, Gordon J, Hassidis G, Komenou A (2001). The enigma of harbour porpoise presence in the Mediterranean Sea. Mar Mamm Sci.

[B18] Pritchard JK, Stephens M, Donnelly P (2000). Inference of population structure using multilocus genotype data. Genetics.

[B19] Falush D, Stephens M, Pritchard JK (2003). Inference of population structure using multilocus genotype data: linked loci and correlated allele frequencies. Genetics.

[B20] Guillot G, Estoup A, Mortier F, Cosson J-F (2005). A spatial model for landscape genetics. Genetics.

[B21] Rousset F (2000). Genetic differentiation between individuals. J Evol Biol.

[B22] Wilson GA, Rannala B (2003). Bayesian inference of recent migration rates using multilocus genotypes. Genetics.

[B23] Rosenberg NA, Pritchard JK, Weber JL, Cann HM, Kidd KK, Zhivotovsky LA, Feldman MW (2002). Genetic structure of human populations. Science.

[B24] Weir BS, Cockerham CC (1984). Estimating F-statistics for the analysis of population structure. Evolution.

[B25] Rosenberg NA, Mahajan S, Ramachandran S, Zhao C, Pritchard JK, Feldman MW (2005). Clines, clusters, and the effect of study design on the inference of human population structure. PLoS Genet.

[B26] Rousset F (1997). Genetic differentiation and estimation of gene flow from F-statistics under isolation by distance. Genetics.

[B27] Slatkin M (1993). Isolation by distance in equilibrium and non-equilibrium populations. Evolution.

[B28] Leblois R, Estoup A, Rousset F (2003). Influence of mutational and sampling factors on the estimation of demographic parameters in a "Continuous" population under isolation by distance. Mol Biol Evol.

[B29] Palumbi SR (2004). Marine reserves and ocean neighborhoods: the spatial scale of marine populations and their management. Annu Rev Environ Resourc.

[B30] Rousset F (2004). Genetic Structure and Selection in Subdivided Populations (Monographs in population biology edn).

[B31] Andersen LW, Ruzzante DE, Walton M, Berggren P, Bjørge A, Lockyer C (2001). Conservation genetics of harbour porpoises, *Phocoena phocoena*, in eastern and central North Atlantic. Conserv Genet.

[B32] Andersen LW, Tromsø: NAMMCO Scientific Publications (2003). Harbour porpoises (*Phocoena phocoena*) in the North Atlantic: distribution and genetic population structure. Harbour Porpoises in the North Atlantic.

[B33] Rosel P, Dizon AE, Haygood MG (1995). Variability of the mitochondrial control region in populations of the harbour porpoise, *Phocoena phocoena*, on interoceanic and regional scales. Can J Fish Aquat Sci.

[B34] Gol'din PE (2004). Growth and body size of the harbour porpoise, *Phocoena phocoena *(Cetacea, Phocoenidae), in the Sea of Azov and the Black Sea. Vestnik Zoologii.

[B35] Tolley KA, Rosel PE (2006). Population structure and historical demography of eastern North Atlantic harbour porpoises inferred through mtDNA sequences. Mar Ecol Prog Ser.

[B36] Koutsikopoulos C, Beillois P, Leroy C, Taillefer F (1998). Temporal trends and spatial structure of the sea surface temperature in the Bay of Biscay. Oceanol Acta.

[B37] Koutsikopoulos C, Le Cann B (1996). Physical processes and hydrological structures related to the Bay of Biscay anchovy. Sci Mar.

[B38] Fiùza AFG, Suess E, Thiede J (1983). Upwelling patterns off Portugal. Coastal Upwelling: Its Sediment Records (part A).

[B39] Tenore KR, Alonso-Noval M, Alvarez-Ossorio M, Atkinson LP, Cabanas JM, Cal RM, Campos HJ, Castillejo F, Chesney EJ, Gonzalez N (1995). Fisheries and oceanography off Galicia, NW Spain: mesoscale spatial and temporal changes in physical processes and resultant patterns of biological productivity. J Geophys Res.

[B40] Southward AJ, Hawkins SJ, Burrows MT (1995). Seventy years' observations of changes in distribution and abundance of zooplankton and intertidal organisms in the western English Channel in relation to rising sea temperature. J Therm Biol.

[B41] OSPAR Commission (2000). Quality Status Report 2000: Region I – Arctic Water.

[B42] Donovan GP, Bjørge A (1995). Harbour porpoises in the North Atlantic: edited extract from the report of the IWC scientific committee, Dublin 1995. Report of the International Whaling Commission.

[B43] Hutchison DW, Templeton AR (1999). Correlation of pairwise genetics and geographic distance measures: inferring the relative influences of gene flow and drift on the distribution of genetic variability. Evolution.

[B44] Leblois R, Rousset F, Estoup A (2004). Influence of spatial and temporal heterogeneities on the estimation of demographic parameters in a continuous population using individual microsatellite data. Genetics.

[B45] Hammond PS, Berggren P, Benke H, Borchers DL, Collet A, Heide-Jorgensen MP, Heimlich S, Hiby AR, Leopold MF, Øien N (2002). Abundance of harbour porpoises and other cetaceans in the North Sea and adjacent waters. J Applied Ecol.

[B46] SCANS-II : Quarterly newsletter for the small cetaceans in the European Atlantic and North Sea project. Special Issue 9: Survey of the SCANS-II project. http://biology.st-andrews.ac.uk/scans2/documents/issue9_Dec06.pdf.

[B47] Longhurst AR (1998). Ecological Geography of the Sea.

[B48] Özsoy E, Ünlüata Ü (1997). Oceanography of the Black Sea: a review of some recent results. Earth Sci Rev.

[B49] Theocharis A, Georgopoulos D, Lascaratos A, Nittis K (1993). Water masses circulation in the central region of the Eastern Mediterranean: Eastern Ionian, South Aegean, and Northwest Levantin 1986–1987. Deep-Sea Res II.

[B50] Moraitou-Apostolopoulou M, Moraitou-Apostolopoulou M, Kiortsis V (1985). The zooplankton communities of the Eastern Mediterranean (Levantine basin, Aegean Sea); influence of man-made factors. Mediterranean Marine Ecosystems.

[B51] Richardson AJ, Schoeman DS (2004). Climate impact on plankton ecosystems in the Northeast Atlantic. Science.

[B52] Sathyendranath S, Cota G, Stuart V, Maass H, Platt T (2001). Remote sensing of phytoplankton pigments: a comparison of empirical and theoretical approaches. Int J Remote Sens.

[B53] Behrenfeld MJ, O'Malley RT, Siegel DA, McClain CR, Sarmiento JL, Feldman GC, Millingan AJ, Falkowski PG, Letelier RM, Boss ES (2006). Climate-driven trends in contemporary ocean productivity. Nature.

[B54] Forney KA (1999). Trends in harbour porpoise abundance off central California, 1986–95: evidence for interannual changes in distribution?. J Cetacean Res Manage.

[B55] Johnston DW, Westgate AJ, Read AJ (2005). Effects of fine-scale oceanographic features on the distribution and movements of harbour porpoises *Phocoena phocoena *in Bay of Fundy. Mar Ecol Prog Ser.

[B56] Carretta JV, Taylor BL, Chivers S (2001). Abundance and depth distribution of harbor porpoise (*Phocoena phocoena*) in northern California determined from a 1995 ship survey. Fish Bull.

[B57] Fontaine MC, Tolley KA, Siebert U, Gobert S, Lepoint G, Bouquegneau JM, Das K (2007). Long-term feeding ecology and habitat use in harbour porpoises *Phocoena phocoena *from Scandinavian waters inferred from trace elements and stable isotopes. BMC Ecol.

[B58] MacLeod CD, Santos MB, Reid RJ, Scott BE, Pierce GJ (2007). Linking sandeel consumption and the likelihood of starvation in harbour porpoises in the Scottish North Sea: could climate change mean more starving porpoises?. Biol Lett.

[B59] Li WKW (2002). Macroecological patterns of phytoplankton in the northwestern North Atlantic. Nature.

[B60] Beaugrand G, Reid PC, Ibanez F, Lindley JA, Edwards M (2002). Reorganization of North Atlantic marine copepod biodiversity and climate. Science.

[B61] Perry AL, Low PL, Ellis JR, Reynolds JD (2005). Climate change and distribution shifts in marine fishes. Science.

[B62] Attrill MJ, Power M (2002). Climatic influence on marine fish assemblage. Nature.

[B63] Learmonth JA, MacLeod CD, Santos MB, Pierce GJ, Crick HQP, Robinson RA (2006). Potential effects of climate change on marine mammals. Oceanogr Mar Biol.

[B64] Duke S (2003). The Population and the Social Structure of Harbour Porpoise (Phocoena phocoena) from Around the Coasts of Iceland and Ireland.

[B65] Fontaine MC, Galan M, Bouquegneau JM, Michaux JR (2006). Efficiency of fluorescent multiplex polymerase chain reactions (PCRs) for rapid genotyping of harbour porpoises *Phocoena phocoena *with 11 microsatellite loci. Aquat Mamm.

[B66] National Oceanographic Data Centre. http://www.nodc.noaa.gov/.

[B67] National Geophysical Data Centre. http://www.ngdc.noaa.gov/products/ngdc_products.html.

[B68] NASASea-viewing Wide Field-of-view Sensor database. http://oceancolor.gsfc.nasa.gov/.

[B69] Guillot G, Mortier F, Estoup A (2005). GENELAND: a computer package for landscape genetics. Mol Ecol Notes.

[B70] Goudet J FSTAT, a program to estimate and test gene diversities and fixation indices (version 2.9.3). http://www2.unil.ch/popgen/softwares/fstat.htm.

[B71] Zar JH (1999). Biostatistical analysis.

[B72] Raymond M, Rousset F (1995). GENEPOP (Version 1.2): population genetics software for exact tests and eucumenism. J Hered.

[B73] Wright S (1943). Isolation by distance. Genetics.

[B74] Hardy OJ, Vekemans X (2002). SPAGeDi: a versatile computer program to analyse spatial genetic structure at the individual of population levels. Mol Ecol Notes.

[B75] Ray N (2005). PATHMATRIX: a geographical information system tool to compute effective distances among samples. Mol Ecol Notes.

[B76] Adriaensen F, Chardon JP, De Blust G, Swinnen E, Villalba S, Gulinck H, Matthysen E (2003). The application of 'least-cost' modelling as a functional landscape model. Land Urb Plan.

[B77] Nelson A, Turner A (2004). Processing global self-consistent hierarchical high resolution shoreline data version 1.2 Into ESRI ArcGIS vector and raster data.

[B78] Wessel P, Smith WHF (1996). A global self-consistent, hierarchical, high-resolution shoreline database. J Geophys Res.

[B79] Rosenberg NA (2004). DISTRUCT: a program for graphical display for population structure. Mol Ecol Notes.

[B80] Feldman GC, McClain CR, Kuring N, Bailey SW, Thomas D, Franz BF, Meister G, Werdell PJ, Eplee RE, MacDonald M, Rubens M (2006). Seasonal, monthly, and weekly climatologies. Ocean Color Web.

