# Supplementary material for: Rise of oceanographic barriers in continuous populations of a cetacean: the genetic structure of harbour porpoises in Old World waters
Source: BMC Biol. 2007 Jul 25;5:30. doi: 10.1186/1741-7007-5-30 (PMC1971045; doi:10.1186/1741-7007-5-30)
Supplement: Additional file 1 — Levels of genetic differentiation at microsatellite loci estimated as FST among the populations inferred from the cluster analyses. The North Atlantic cluster (NAt) was subdivided latitudinally in three parts (see Figure 1). The FST values [95% CI] are below the diagonal and the significance level of the exact tests for population differentiation [72] are above. [file 1741-7007-5-30-S1.pdf]

Additional File 1: Levels of genetic differentiation at microsatellite loci estimated as F_{ST} among the populations inferred from the cluster analyses

	Black Sea	Iberia	North Atlantic cluster		
			NAt-3A	*NAt-3B*	*NAt-3C*
Black Sea	-	<0.0001	<0.0001	<0.0001	<0.0001
Iberia	0.314 [0.240 - 0.381]	-	<0.0001	<0.0001	<0.0001
North Atlantic cluster					
NAt-3A	0.153 [0.118 - 0.191]	0.085 [0.049 - 0.124]	-	0.0123	<0.0001
NAt-3B	0.160 [0.120 - 0.201]	0.095 [0.056 - 0.142]	<0.001 [<0.001 - <0.001]	-	<0.0001
NAt-3C	0.154 [0.124 - 0.186]	0.097 [0.059 - 0.139]	0.001 [<0.001 - 0.002]	<0.001 [<0.001 - 0.001]	-

The *North Atlantic* cluster (*NAt*) was subdivided latitudinally in three parts (see Figure 1). The F_{ST} values [95% CI] are below the diagonal and the significance level of the exact tests for population differentiation [72] are above.
